# Scirrhous Cancer of the Rectum in a Boy Eighteen Years of Age

**Published:** 1883-06

**Authors:** Herman Hayd

**Affiliations:** England


### Clinical Reports.

### Scirrhous Cancer of Rectum in a Boy 18 Years of Age.

By Herman Hayd, M. R. C. S., England.

A few weeks ago, I was called to one of the neighboring towns to see a young man supposed to be suffering from chronic diarrhœa. He was pale, anemic, and very much emaciated, and sat with his legs elevated above his head upon his tuber ischii. His face was drawn and anxious and indicative of much suffering. The clinical history is briefly as follows:

About one year and a half ago, he commenced to complain of some uneasiness, which at times amounted to slight pain upon stool, and he occasionally noticed streaks of blood in the motions, when his bowels were constipated. This condition continued, but yet attracted very little attention, until six months ago, when he was suddenly seized with a violent and nearly fatal hemorrhage. The bleeding was arrested, he was confined to his bed a few days, and then resumed his work, apparently feeling quite well. But the pain upon stool increased, repeated hemorrhages occurred, attacks of constipation, alternately with diarrhœa, accompanied with great tormina, and tenesmus set in, making these last few months of life wretched and miserable; no bladder symptoms. Upon palpation over the ischiorectal region, a deep, firm, hard mass was to be felt, firmly adherent to the adjacent skin and cellular tissue. Protruding through the anal orifice was to be seen two or three small, but very hard masses, nodules, resembling a healed or thrombosed hemorrhoid. Chloroform was administered and the rectum thoroughly examined. The sphincters were found to be much thickened and infiltrated, although still possessing contractile power. After passing through the internal sphincter, the whole rectum seemed to be one mass of cancer, thickened, immovable, and all over studded with prominent, hard, cartilaginous, irregular nodules.

About the junction of the second and third divisions of the rectum, was a very definite structure, with irregular, rough and infiltrated margins, with an opening large enough to admit the index finger. Above this structure the bowel was very much dilated, fixed, and apparently infiltrated up to the sigmoid flexure. Upon removing the finger at least half a pint of a dirty, greenish, semi-gelatinous, stinking fluid escaped. By deep palpation over the left iliac region, a resisting mass could be felt, and the mesenteric and lumbar glands were found to be enlarged. An interesting feature about the case was this, that notwithstanding the large size of the growth and the involvement of the abdominal lymphatic glands, still, those in the groin along poupart's ligament, and beneath it, and those in Scarpa's triangle were unaffected. Here clinical observation demonstrates what we know to be anatomical facts, and points out for us the course of the lymphatic vessels. Had the growth involved the penis, the inguinal glands would have been enlarged as the lymphatic vessels of the penis empty into these glands; while on the other hand a malignant growth of the rectum does not involve these external glands until the cutaneous surface or superficial parts of the perinæum or scrotom are affected. The treatment suggested was only palliative, abundance of nutritious and easily assimilated food, gentle laxatives and morphia " ad libitum," to soothe pain, and make a most miserable life as free from pain and suffering as possible. I advised that the nurse should have beside her a hypodermic syringe, and inject a quarter of a grain of morphia, or more, just before a motion of the bowels was expected, and thus lessen the excruciating agony suffered by these patients at this time. Lumbar colotomy was deemed impracticable. This case appears to me to be a most instructive one, and from which some practical deductions can be drawn. *First.* Let us not permit ourselves to treat a so-called chronic diarrhœa or dysentery for months without repeatedly examining the lower bowel, and if necessary with the assistance of an anæsthetic. *Second.* Let us not be too sanguine of the possible non-existence of malignant tumors during youth and adolescence, for autopsies have upon several occasions revealed cancerous masses in various organs, even in very young children. *Third.* Let us not think that a history of heredity is always necessary before we can feel satisfied with a diagnosis of cancer or other allied affections, because one sees many cases of cancer and tubercle where no such history can be obtained. *Fourth.* For any suspicious tumor operate early, and if it returns operate a second or third time, because many of the semi-malignant or sarcomatous tumors seem to lose their malignancy after repeated extirpation. Of course the converse is sometimes equally true, that they seem to recur with increased malignancy.

Much might be said upon the etiology of malignant growths in general, but I shall leave this part of my subject for a future communication.

